# Evaluation and impact factors of international competitiveness of China’s cobalt industry from the perspective of trade networks

**DOI:** 10.1038/s41598-024-63104-w

**Published:** 2024-05-28

**Authors:** Ligang Xu, Xiang Guo, Meijuan Xu, Yanglei Jia, Zhengfang Zhong

**Affiliations:** 1 Mining Development Research Center, Jiangxi University of Science and Technology, Ganzhou, 341000 China; 2 School of Economics Management, Jiangxi University of Science and Technology, Ganzhou, 341000 China; 3 School of Economics and Management, Fuzhou University, Fuzhou, 350108 China; 4 Business School, Monash University, Melbourne, VIC 3163 Australia

**Keywords:** Environmental social sciences, Applied mathematics

## Abstract

In recent years, with the development of the new energy industry, the demand for cobalt as a raw material for power batteries has been increasing. However, China itself has a shortage of cobalt resources. Therefore, overcoming poor resource conditions and enhancing the international competitiveness of the cobalt industry have become urgent issues. This paper is based on global trade data on cobalt resources from 2007 to 2020. A panel regression model is constructed from the perspective of trade networks, and Entropy-Topsis is used to construct a comprehensive evaluation index system for the international competitiveness of critical nonferrous metals. This study empirically examines the impact of the trade network characteristics of cobalt resources on international competitiveness, assigns practical significance to trade network characteristic indicators, and analyses the overall competitiveness changes in the global cobalt industry chain and its upstream, midstream, and downstream sectors. The research findings reveal the following key points: (1) In recent years, the competitive focus of the cobalt industry chain in various countries has shifted from upstream and midstream to midstream and downstream, with increasingly fierce trade competition downstream, gradually tilting toward countries such as South Korea, Japan, and China. (2) Cobalt trade competition, which was initially characterized by competition among multiple countries, has gradually become more centralized and stable, with differences in the competitiveness of various countries occurring at different stages of the cobalt industry chain. (3) Network centrality and network heterogeneity both have a significant promoting effect on the international competitiveness of the industry, while network connectivity has a significant inhibitory effect on the improvement of international competitiveness.On this basis, the study also suggests some policy implications. The purpose of the study is to enhance the international competitiveness of China's cobalt industry from a trade perspective and to investigate the developments of cobalt trade between China and the rest of the world.

## Introduction

With the industrial changes and technological advances of the twenty-first century, the pattern of international supply and demand for resources, as well as the direction of trade flows, has been greatly affected^1–3^. The intricate interplay among governments, organizations, transnational corporations, and other economic entities has given rise to a resource flow pattern known as a "regional bloc," which has, to some extent, impeded the development and utilization of resources in different countries^4,5^. Enhancing the international competitiveness of domestic industries in the face of complex trade competition is a crucial focus for future research on resource flows in the context of globalization^6–8^. Cobalt is regarded as a strategic raw material due to its importance in key industries. The United States, Japan, Australia, and other regions have included cobalt in their lists of critical minerals. Currently, the advancement of strategic emerging industries, such as new energy vehicles, relies heavily on the utilization of cobalt resources^9–12^. China, being a major producer of refined cobalt, has low reserves of cobalt resources, with a high foreign dependence rate of 97%. This results in a lack of pricing power in the cobalt market. Given the current technological and pricing conditions, China's cobalt resources depend on imports through foreign trade to meet domestic market demands, leading to a significant increase in supply risk^13–15^. In the past two years, the global cobalt supply has been affected by the impact of the covid19, leading to increased instability. As a result, the cobalt industry has maintained low inventory in the short term to balance supply and demand, while long-term supply continues to rely on the commissioning of new mining projects^16^. Despite the current global emphasis on "cobalt-free" alternatives, the limited substitutability of cobalt resources has led to an expansion in global total consumption demand. Consequently, urgent attention is required to enhance China's cobalt resources industry's international competitiveness and to identify the key factors affecting its development.

## Literature review

Currently, the primary focus for improving the international competitiveness of China's nonferrous metal industry is centered on optimizing the utilization of trade advantages. The concept of trade advantage refers to a favorable situation or condition that facilitates gains in international trade. The theory of trade advantage primarily encompasses the theories of comparative advantage and competitive advantage. This paper organizes the research of related scholars in two ways.

The first is a study on the methodology for measuring the international competitiveness of industries. Currently, assessments of industries' international competitiveness can be divided into two main approaches. The first approach involves the use of a single indicator, such as the international market share indicator (IMS), the revealed comparative advantage indicator (RCA), the net revealed comparative advantage indicator (NRCA), the trade competition indicator (TC), productivity, market share, and profitability, among others, to measure the international competitiveness of industries^17–19^. The second method involves utilizing a range of indicators to establish a comprehensive evaluation index system for assessing the international competitiveness of industries^20,21^. The comprehensive evaluation index system for assessing the international competitiveness of industries can also be divided into two main types. The first type involves constructing the index system based on the "diamond model" and its derivatives^22^. The second type involves constructing an index system based on the theory of comparative advantage and the theory of competitive advantage and selecting different comparative advantage indicators and competitive advantage indicators^23–25^. This approach allows for the observation of the international competitiveness status of industries and its changes from multiple perspectives. However, a limited number of extant studies have comprehensively assessed the international competitiveness of industries based on theories of comparative advantage and competitive advantage. The practice of using Relative Comparative Advantage (RCA) indicators to measure international competitiveness has been widely adopted by academics. However, there are dissenting voices arguing that in the context of today's global value chain division of labor, a single RCA indicator cannot accurately capture competitiveness^26,27^. Therefore, we draw upon existing studies^20,28,29^ and employ Entropy-Topsis to construct a comprehensive evaluation index system for assessing the international competitiveness of industries from a trade perspective.

The second is a study on the enhancement of the international competitiveness of industries. The enhancement of industrial international competitiveness encompasses various factors, including the expansion of market demand, changes in production factors, enhancement of the competitive environment, reduction of tariffs and increase in subsidies, and policy support, among others^30–32^. However, traditional international trade theories face challenges in explaining the complexities of current global trade multilaterals. The expansion of supply chains and trade in intermediate goods has led to the development of an intricate trade network characterized by interaction, mutual influence, and interdependence among countries^33,34^. Conventional multivariate statistical analysis is infrequently employed for the analysis of correlation data, as it is challenging for correlation data to meet the "variable independence hypothesis" in the traditional statistical context. From the standpoint of trade networks, employing complex network analysis to examine the trade connections between nations can render the original bilateral trade interactions within a more intricate international trade network topology. This approach can more comprehensively depict the network characteristics and position of a country within the international trade network^35,36^. Consequently, this study utilizes the characteristics of the trade network to develop a panel regression model for assessing the extent of trade through network centrality, the volume of trade through network connectivity, and the quality of trade through network heterogeneity. We also propose multidimensional research hypotheses to empirically examine the impact of various trade network characteristics on the international competitiveness of industries.

Compared to previous literature, this study makes three main contributions: (1) Given the practical significance of indicators of trade network characteristics: Previous studies have only considered the impact of various factors on the international competitiveness of industries based on their individual trade, without considering the intricate network created by the multilateral trade among different countries. This study adopts the perspective of trade networks and explores the intrinsic connection between various trade network characteristics and the international competitiveness of industries through the construction of a panel regression model. It expands the application of the complex network method, providing trade characteristic indicators with practical significance for countries to enhance the international competitiveness of crucial nonferrous metal industries. Additionally, the study considers the impact of trade network characteristics on the international competitiveness of industries. (2) Analyzed the Evolution of Competitiveness: Among the existing studies, there are fewer analyses that utilize the theory of comparative advantage and the theory of competitive advantage to perform a comprehensive evaluation of the international competitiveness of the cobalt industry. However, this study comprehensively considers the special background of cobalt resources, selects appropriate comparative advantage indicators and competitive advantage indicators, constructs a comprehensive evaluation index system for the international competitiveness of industries, and measures the comprehensive index based on the import and export trade data of global cobalt resources, in order to empirically study the changes in the international competitiveness of the global cobalt industry chain as a whole, as well as the upstream, midstream and downstream industries. (3) Provides policy insights for improving the international competitiveness of China's cobalt industry: Based on the trade perspective, policy approaches have been proposed, including enhancing the depth and breadth of trade, extending the industrial chain, diversifying the cobalt supply, and ensuring the security of raw material supply.

## Theoretical basis and research hypothesis

### Network centrality and industrial international competitiveness

Network centrality is indicative of a node's control and position within a network^37^, and it reflects the breadth of a country's trade. In the context of the international trade network, a higher network centrality for a country signifies a more central role in trade, with increased trade transactions with influential countries. This finding suggests that other trading nations have a degree of trade dependence on the country, while also indicating that the country has greater control and a higher status within the network. Consequently, this paper presents the following hypotheses:

#### Hypothesis 1

Within the cobalt resources trade network, the greater the centrality of a country in the network is, the more advantageous it is for enhancing that country's industrial international competitiveness.

### Network connectivity and industrial international competitiveness

The concept of network connectivity encompasses the proximity, longevity, frequency, and extent of connections between network nodes, serving as an indicator of the proximity of links between individual nodes and other nodes within the network^38^. The traditional theory of comparative advantage posits that countries engage in reciprocal trade, whereby the larger a country's trade volume with other trading partners is, the more it can acquire a greater market share and a stronger capacity for resource allocation. Simultaneously, this process can enhance the country's technological endowment through the technology spillover effect. Nevertheless, an excessive market share of cobalt, a strategically important metal resource, may not be beneficial for enhancing the international competitiveness of the industry. There are three primary explanations for this phenomenon. First, trade transactions are contingent upon the bilateral relationships between the involved countries, and the pressure of resource allocation may hinder the efficient allocation of resources to establish extensive connections with other nations. Second, the transfer of high-quality technology relies on the trust relationship between the investing country and the resource-rich country. However, cultural and political disparities in resource-rich countries pose challenges in deepening the level of trust and reaping the benefits of technological spillover. Finally, owing to the nonrenewable nature of resources and high transportation costs, resource-exporting nations often occupy lower rungs of the industrial chain, exporting low-grade products that lack competitiveness. Consequently, hypothesis 2 is as follows:

#### Hypothesis 2

In the cobalt resource trade network, the greater the network connectivity of a country is, the less favorable it is for enhancing that country's industrial international competitiveness.

### Network heterogeneity and industrial international competitiveness

In complex networks, network heterogeneity refers to the presence of nonredundant connections between nodes in a network and may indicate the presence of structural holes or weak connections between networks^39^. A higher level of heterogeneity suggests that nodes can access diverse information from various sources, enabling them to maintain a competitive advantage. For the network as a whole, a greater number of structural holes indicates the presence of more crucial nodes in the network and greater redundancy of information between the nodes. Consequently, the network structure is more significantly influenced by the important nodes, leading to increased overall instability. Regarding the nodes, the primary sender of information can assume a specific control position within the network. Additionally, in the event of a complete network collapse, the local network can be stabilized. In the context of the international trade network, network heterogeneity indicates the level of geographic concentration of national resource trade. A lower degree of geographic concentration implies a more dispersed distribution of trade partners across different countries. Hence, within the cobalt resources trade network, it is advantageous for trade relations between different countries to be nonredundant. On the one hand, a high level of network heterogeneity in a country implies the ability to engage in direct trade with a larger number of countries, facilitating the identification of high-quality trading partners, providing opportunities for technology and personnel acquisition through the imitation effect, and enabling better integration of the country's advantages into the international division of labor. This contributes to the country's enhanced position in the global value chain, thereby strengthening the international competitiveness of its industry. On the other hand, increased network heterogeneity suggests diversified trade partners and a more stable trade network, reducing susceptibility to external influence in foreign trade. This can effectively mitigate the resource risk associated with trade monopolies held by oligopolistic countries. Based on this, hypothesis 1 is proposed:

#### Hypothesis 3

In the cobalt resource trade network, a greater degree of network heterogeneity within a country is positively associated with the enhancement of that country's industrial international competitiveness.

## Methodology

### Variable and calculation instructions

#### Explained variables

The concept of industrial international competitiveness lacks a precise definition in economics. Porter, an early scholar in the field, has studied international competitiveness at the industrial level and defined it as the ability of a country to establish a conducive business environment that enables its enterprises to gain a competitive advantage^40^. This paper primarily focuses on the strategic position of critical nonferrous metals in international trade and aims to explore how to leverage "trade advantage" from a research perspective. Hence, we define international competitiveness in critical nonferrous metal industries as a country's import and export capacity to compete in the global market and gain trade benefits through competition.

The cobalt resource industry chain can be categorized into three levels: upstream, midstream, and downstream. The upstream products primarily consist of cobalt ore, which serves as a fundamental raw material. The midstream products mainly comprise cobalt chemical products, including cobalt oxides and electrolytic cobalt, which are utilized as cathode materials for lithium batteries and in the production of magnetic materials. The downstream products predominantly encompass cobalt, with lithium cobalt oxide being utilized in the 3C battery field. We examined the "Cobalt" products listed in the United Nations Commodity Trade Statistics Database (UN Comtrade) from 2007 to 2020. Our selections included upstream cobalt ores and concentrates (HS code: 260500), midstream cobalt tetraoxide (HS code: 282200), and electrolytic cobalt (HS code: 81052). The study focused on the midstream product (HS code: 810520) and the downstream product lithium cobalt oxide (HS code: 284190). National trade data for each product were obtained from UN Comtrade, and the industry's international competitiveness was assessed using a comprehensive index based on these data.

As shown in Table 1, this study utilizes the 2007 International Competitiveness Composite Index of the U.S. cobalt resource industry as the base period based on the foundational evaluation indicators mentioned above. The indices for the A and B categories for the year 2008 are calculated using Formula 1 and Formula 2. This process is repeated to estimate the comprehensive international competitiveness indices of cobalt resource industries for various countries globally from 2007 to 2020.

1 $${\text{Indicator}}\;{\text{A}}:\;{\text{An}}\;{\text{index}}\;{\text{for}}\;{2}00{8} = \left( {{\text{A}}\;{\text{in}}\;{2}00{8} - {\text{A}}\;{\text{in}}\;{\text{the}}\;{\text{United}}\;{\text{States}}\;{\text{in}}\;{2}00{7}} \right) + {1}00$$

2 $${\text{Indicator}}\;{\text{B}}:\;{\text{An}}\;{\text{index}}\;{\text{for}}\;{2}00{8} = \left( {{\text{B}}\;{\text{in}}\;{2}00{8} \div {\text{B}}\;{\text{in}}\;{\text{the}}\;{\text{United}}\;{\text{States}}\;{\text{in}}\;{2}00{7}} \right) \times {1}00$$

**Table 1:** The evaluation index system for international industrial competitiveness.

Target layer	Factor layer	Fundamental evaluation Indicators
International competitiveness of industries (CI)	Competitive advantage	Average International Market Share(AMS)
		Trade Competitive advantage index (TC)
	Comparative advantage	Industrial Export Proportion index (EP)
		Revealed comparative advantage index (RCA)

Among these indicators, indicator A comprises TC, while indicator B comprises AMS, EP, and RCA. Due to the varying degrees of variation exhibited by each indicator, Entropy-TOPSIS was employed to adjust the weights of each indicator, thereby deriving the indicator weights and measurements^28,29^.The advantages of choosing Entropy-Topsis are mainly reflected in the following three points: (1) The classical Topsis method assigns the same weights to each indicator by default, which may influence the results to a certain extent. In contrast, the use of Entropy-Topsis can reduce the subjective assignment of weights, thereby minimizing bias; (2) Entropy-Topsis, in comparison to other methods, transforms numerical values into a sorted evaluation, making it more convenient for the comprehensive evaluation of the international competitiveness of industries; (3) Through Entropy-Topsis, multiple basic indicators are integrated, making it more suitable for reflecting the comprehensive level of the research object, especially when it is characterized by complexity. The specific steps: Firstly, the measured values of the basic evaluation indexes are standardized. Secondly, the entropy method is used to calculate the weights of the indexes. Furthermore, the data, after multiplying the weights and the standardized data, is used as the Topsis raw data. Finally, the Topsis is used to calculate the Composite Index of International Competitiveness of the industry (CI), and the measured values are analyzed.

The specific operational steps are as follows: (1) Standardize the measured values of the basic evaluation indicators mentioned above using SPSS software.(2) Calculate the weight of each indicator using the entropy value method; (3) Multiply the weights by the standardized data to obtain Topsis raw data. (4) Calculate industry's international competitiveness composite (CI) using with Topsis and analyze obtained measured values.

(1)Average International Market Share (AMS).

The average international market share (AMS) is the percentage of a country's exports of a specific product in the global market of a particular region. This index is designed to address regional issues that exist within the market share (MS) index and serves as a valuable indicator of the industry's competitive advantage. The calculation formula is as follows:

3 $${AMS}_{ijt}=\frac{{E}_{ijt}/{n}_{jt}}{{E}_{wjt}}$$

where ${AMS}_{ijt}$ denotes the mean global market penetration of $j$ commodities within country $i$ in year $t$, ${E}_{ijt}$ represents the value of export trade in country $i$ for product $j$ in year $t$, ${E}_{wjt}$ represents the total trade volume of $j$ types of products exported globally in year $t$, and ${n}_{jt}$ represents the total number of countries involved in the export of $j$ types of products in year $t$. Greater values of the AMS index signify a more robust competitive export advantage for products within that particular industry.

(2)Trade competitive advantage (TC) index.

The Trade Competitive index (TC) represents the ratio of a country's net exports of a specific product to the total trade of that product. This index takes into account the impact of re-export trade on export competitiveness and addresses the influence of factors such as re-export trade that are present in market share indices. Its calculation formula is as follows:

4 $${TC}_{ijt}=\frac{{E}_{ijt}-{I}_{ijt}}{{E}_{ijt}+{I}_{ijt}}$$

${TC}_{ijt}$ is the trade competitiveness index of country $i$ for product $j$ in year $t$, ${E}_{ijt}$ is the total export volume of $j$ products of country $i$ in year $t$, and ${I}_{ijt}$ is the total trade imports of country $i$ for product $j$ in year $t$. The TC index ranges from − 1 to 1. A positive value indicates that a country's trade in a specific product is primarily export oriented, with higher production efficiency than the international average and strong export competitiveness. A value close to 0 suggests that the country's trade in the product is mainly intraindustry, prompting some scholars to propose using a country's import and export relative unit prices for similar products to measure the level of intraindustry trade. Conversely, a negative value reflects that the country's trade in the product is primarily import-based, with relatively weak export competitiveness. A TC index closer to 1 signifies stronger international competitiveness.

(3) Industrial export proportion index (EP).

The industrial export proportion index (EP) is a measure of the proportion of a country's exports of a specific product in relation to the country's total export trade volume of all products. This index serves as an indicator of the industry's competitiveness within the country's export market and provides insight into the country's industrial trade. The calculation formula for the EP index is as follows:

5 $${EP}_{ijt}=\frac{{E}_{ijt}}{{E}_{it}}$$

In this formula, ${EP}_{ijt}$ represents the export share of $j$ products in country $i$ in year $t$, ${E}_{ijt}$ denotes the export trade volume of $j$ products in country $i$ in year $t$, and ${E}_{it}$ represents the total export trade of country $i$ in year $t$. The larger the value of the EP index is, the greater the position of the industry among all the industries exporting in the country.

(3) Revealed comparative advantage index (RCA).

The Revealed Comparative Advantage index (RCA) represents the share of a country's total exports of a single product in relation to its overall exports, compared to the global share of that product's exports in relation to total global exports. By excluding fluctuations in national and global totals and considering economic scale, this index ensures the accuracy of a country's export competitiveness between nations. It effectively demonstrates a country's trade advantage in the export of a single product and addresses the issues associated with market share indices. Consequently, it is commonly utilized to reflect a country's international competitiveness with respect to a specific product. The calculation formula for the RCA index is as follows:

6 $${RCA}_{ijt}=\frac{{E}_{ijt}/{E}_{it}}{{E}_{wjt}/{E}_{wt}}$$

${RCA}_{ijt}$ denotes the revealed comparative advantage of product $j$ from country $i$ in year $t$, ${E}_{ijt}$ shows the value of export trade in country $i$ for product $j$ in year $t$, ${E}_{wjt}$ represents the export trade value of the products $j$ in year $t$ on a global scale, and ${E}_{wt}$ is the total export trade volume of the world in year $t$. Generally, an RCA value of less than 0.8 indicates relatively weak competitiveness of a particular industry in a country, while a value between 0.8 and 1.25 suggests a moderate level of export competitiveness. A value between 1.25 and 2.5 indicates strong export competitiveness, and when RCA is equal to or greater than 2.5, the industry in that country has a very strong competitive advantage in the international market.

#### Explanatory variables

First, drawing on existing studies^41^, we use Gephi software to construct a directed weighted network of typical product trade in each stage of the global cobalt industry chain from 2007 to 2020 based on the center theory of complex networks.

Second, each network characteristic index is measured to analyze the influence of a nation's cobalt resource trade network features on the global competitiveness of its sector.

*The network centrality*. The network centrality features are measured using the metric of weighted eigenvector centrality ($InWEigcentrality$). The weighted eigenvector centrality is a measure of the importance of a country or region in a trade network. It takes into account the influence of neighboring trading countries and regions. A higher value indicates a greater radiating influence in the entire trade network. The formula is as follows:

9 $$AX=\lambda X$$

10 $$\lambda X={a}_{1j}\cdot {x}_{1}+{a}_{2j}\cdot {x}_{2}+\cdots +{a}_{ij}\cdot {x}_{i}+\cdots +{a}_{nj}\cdot {x}_{n}\left(i\ne t\right)$$

11 $${C}_{\left(e\right)i}={\lambda }_{i}$$

where $A$ is the $n\times n$ adjacency matrix of one composed of ${a}_{ij}$. $X=\left({x}_{1}, {x}_{2},{ x}_{3}\cdots , {x}_{n}\right)$ denotes the degree centrality of each individual node. ${\lambda }_{i}$ is the eigenvector centrality value. ${a}_{ij}$ represents the degree to which node $i$ contributes to node $j$. ${C}_{\left(e\right)i}$ signifies the centrality of the $i$-node, calculated using weighted eigenvector centrality.

*Network connectivity*. The network connectivity characteristic index is measured by the out-strength ($InWoutdegree$). The out-strength is the total amount of a country's export trade flows to other countries in the network, reflecting the intensity of the country's export trade to other countries and highlighting the extent to which the country is integrated into the global trade network^42^. It is calculated using the following formula:

12 $${S}_{i}^{out}=\sum_{j}^{n}{w}_{ij}$$

where ${S}_{i}^{out}$ denotes the outstrength of node $i$ in the trade network and ${w}_{ij}$ represents the weight of the edge between nodes $i$ and $j$.

*Network heterogeneity*. Network heterogeneity was quantified using the weighted effective size degree ($Ineffecsize$). The weighted effective scale degree is a measure of the proportion of a country's trading contacts with other nations in the network compared to the total number of trade links. A larger effective size indicates greater heterogeneity in the trade network. The formula used for computation is:

13 $${D}_{i}^{e}=\sum_{j}^{n}\left(1-\sum_{q}^{n}{p}_{iq}{m}_{jq}\right), \left(i\ne q,j\right)$$

where ${p}_{iq}$ represents the ratio of trade relations between country $i$ and country $q$ and ${m}_{jq}$ is the marginal strength of the trade relationship between country $j$ and country $q$ (the share of country $j$'s trade with country $q$ in country $q$'s largest trade with other countries).

#### Manipulated variables

Foreign direct investment (FDI): FDI plays a significant role in shaping competitiveness, as it can either facilitate or inhibit GDP growth and development. Foreign direct investment (FDI) leverages a country's inexpensive labor and resource endowment to alleviate financial strain while also introducing advanced knowledge and technology to enhance the country's trade competitiveness. According to the theory of endogenous growth point of view, this is often manifested in the "spillover effect" of knowledge or technology^43^. However, an overabundance of foreign capital may result in foreign entities dominating the host country's consumer market, impeding the country's product trade with other nations^44^.

Level of financial development: The development of an industry cannot be separated from the level of financial services that accompany it. The level of financial development can significantly contribute to a country's international trade through trade structure effects, trade broadening effects, and increased trade complexity. The level of financial development is expressed in terms of private sector domestic credit as a share of GDP^45^.

GDP growth level: The level of GDP growth plays a crucial role in influencing the foreign trade of a country or region^46^. GDP growth has the potential to stimulate trade expansion, thereby enhancing the international competitiveness of domestic industries. Several scholars have analyzed the connection between GDP growth and competitiveness in terms of temporal and spatial dimensions. In the temporal dimension, there exists an unstable declining-curve adaptive relationship between GDP growth and competitiveness, while in the spatial dimension, there is a corresponding relationship between GDP growth and competitiveness^47^.

Trade openness: A country with greater trade openness tends to have fewer trade barriers and a greater level of trade freedom. This can lead to improved efficiency in resource allocation, the promotion of technological progress, and the generation of economies of scale through the learning effect. Simultaneously, engaging in international competition can stimulate industrial economic growth by creating competitive pressure. The degree of external dependence is frequently utilized as a measure of trade openness, specifically, the ratio of a product's import and export trade volume to its GDP^48^.

### Model settings

Based on theoretical foundations and research hypotheses, an econometric model is developed by drawing on the studies of Allen^49^, Nathan^50^, and others.

14 $$InCI={\lambda }_{i}+{p}_{t}+{\alpha }_{1}{InWeigcentrality}_{it}+{\alpha }_{2}{InWoutdegree}_{it}+{\alpha }_{3}{Ineffecsize}_{it}+{\beta }_{1}Infintinvetment$$

$$+{\beta }_{2}Indomprisector+{\beta }_{3}InGDPgrow+{\beta }_{4}InExdependency+{\varepsilon }_{it}$$

In this context, $$i$$, $$t$$, and $$j$$ represent the country, time, and product, respectively. $${\lambda }_{i}$$ denotes the time fixed effect, a specific determinant that can influence the overall competitiveness of the industry and remains consistent over time. $${p}_{t}$$ represents the individual fixed effect, which is a specific factor that can influence the international competitiveness of industries but does not vary with changes in country or region. $${\varepsilon }_{it}$$ is a random disturbance term. $$\alpha$$ represents the explanatory variable, while $$\beta$$ represents the control variable. $${x}_{it}$$ denotes the network characteristic indicators of a country or region, including measures of network centrality ($$InWeigcentrality)$$, network connectivity strength ($$InWoutdegree$$), and network heterogeneity ($$Ineffecsize$$). The other variables are considered control variables: foreign direct investment ($$Infintinvetment$$), the level of financial development ($$Indomprisector$$), the level of GDP growth ($$InGDPgrow$$), and trade openness ($$InExdependency$$).

### Data source

In summary, this study obtained a sample of 871 observations from countries involved in the global cobalt trade between 2004 and 2020 and calculated the numerical values of all variables in the model. All the variables were measured using their natural logarithms. In this case, some of the variables contain negative numbers, and we use data shifting to add 10 to each data to make it positive and then take the natural logarithm. The original data for import and export trade were obtained from the UN Comtrade database, while data on FDI, financial development level, GDP growth level, and trade openness were sourced from the World Bank (WDI) database. The data used to construct the trade network were subjected to descriptive statistical analysis using Stata 15.1, and the results are presented in Table 2.

**Table 2:** Descriptive statistics of each index.

Variable	Mean	Standard deviation	Maximum	Minimum
$InCI$	1.000	0.000765	1.005	1
$InWeigcentrality$	− 2.334	1.103	− 0.126	− 6.969
$InWoutdegree$	− 1.546	1.142	0.683	− 5.013
$Ineffecsize$	1.300	0.392	2.108	0.165
$Infintinvetment$	0.440	0.490	1.937	− 2.777
$Indomprisector$	1.752	0.491	2.376	0
$InGDPgrow$	0.375	0.370	1.401	− 1.917
$InExdependency$	− 4.665	0.865	− 2.793	− 9.252

## Results

### Analysis of comprehensive evaluation indicators for international competitiveness

**Figure 1:**
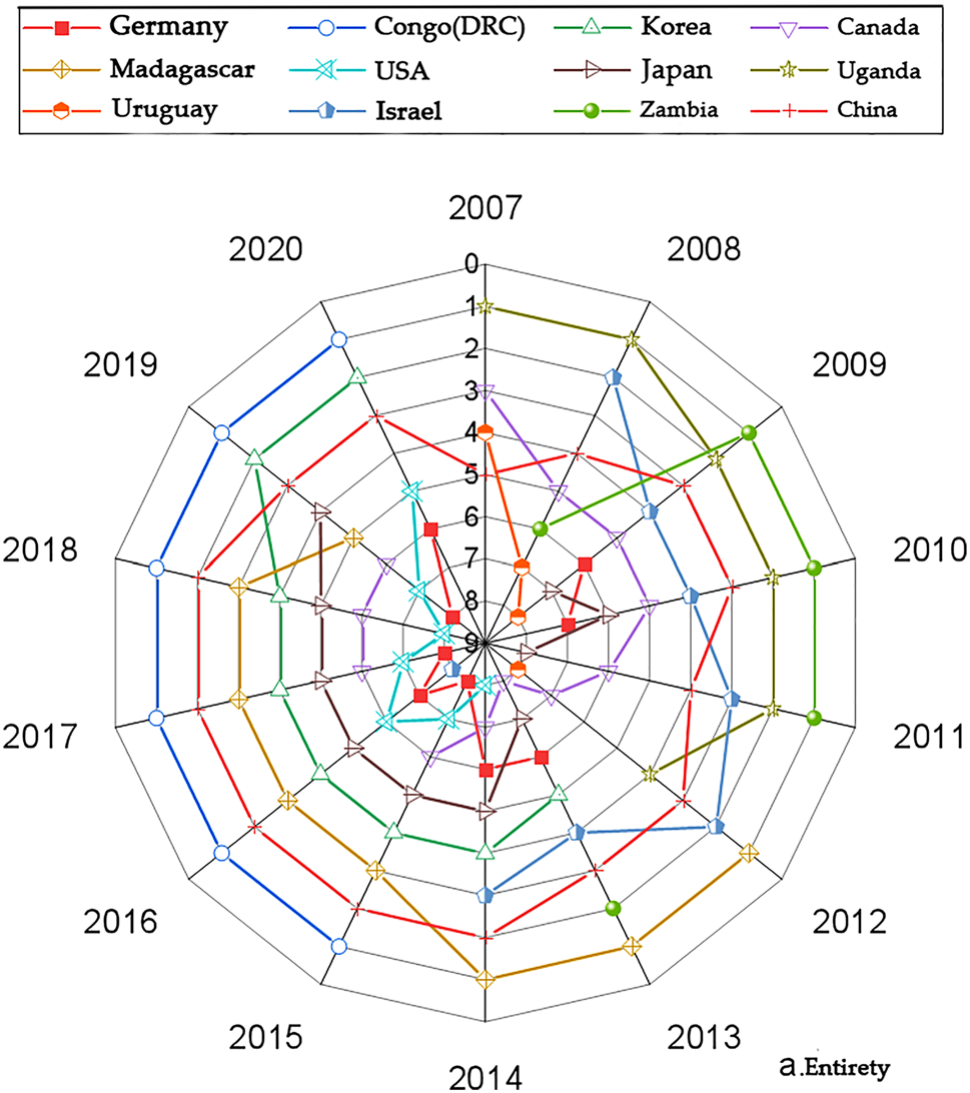
Changes in the entirety CI ranking.

**Figure 2:**
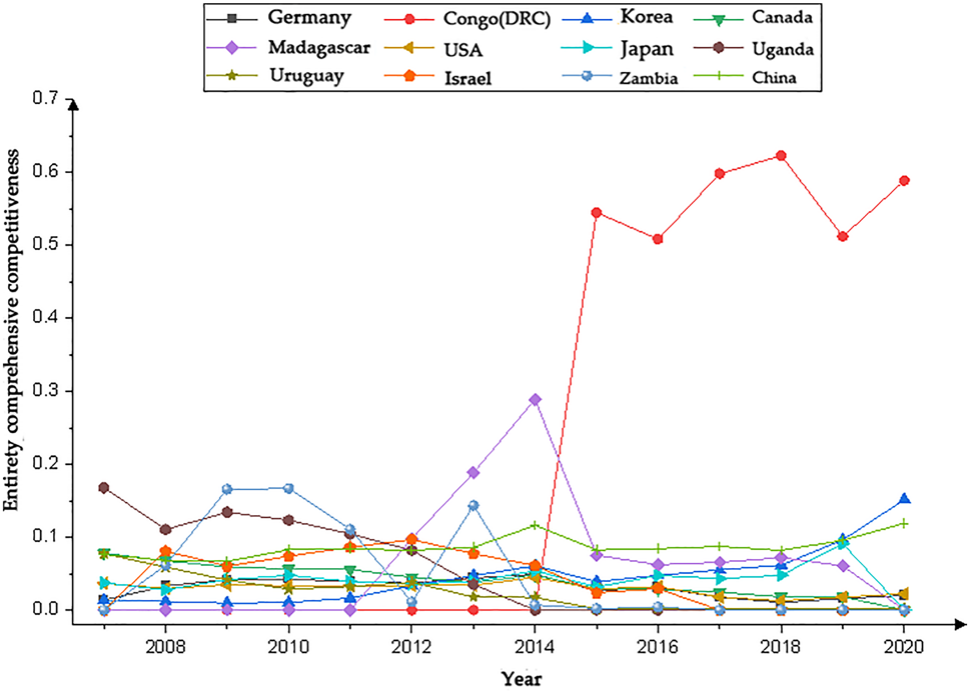
Changes in the entirety CI value.

**Figure 3:**
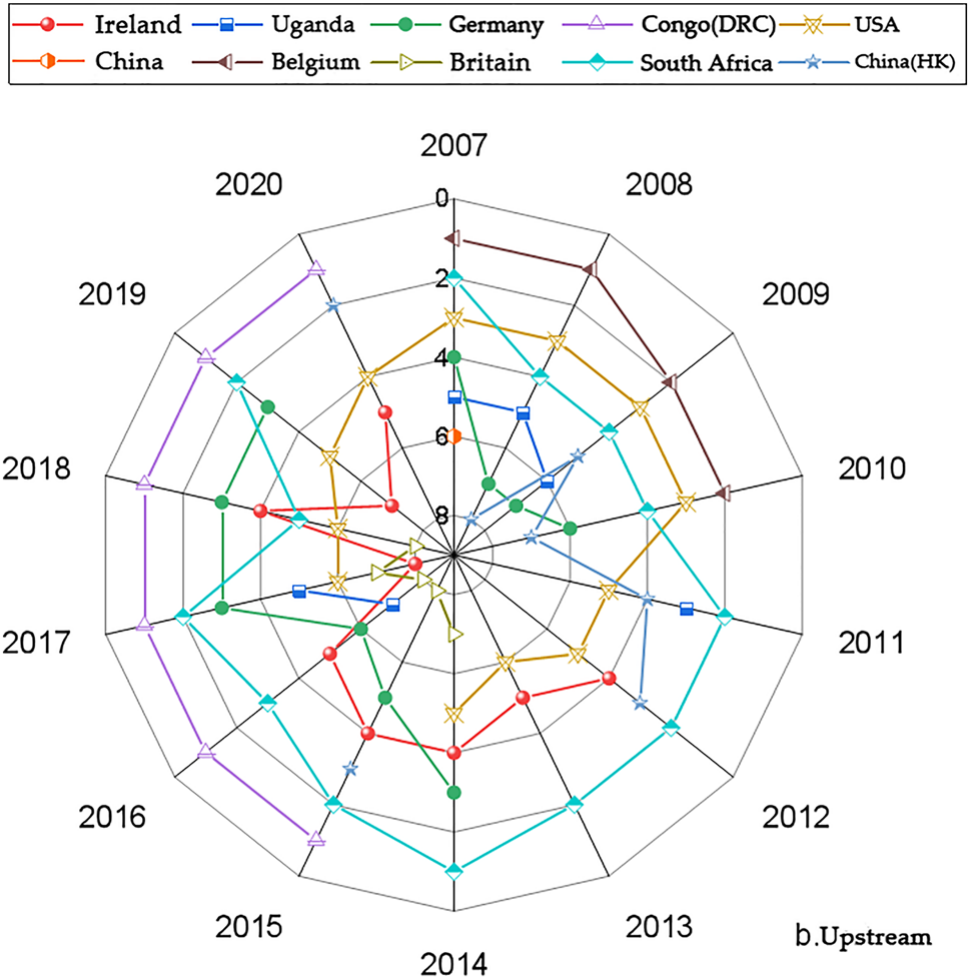
Changes in the upstream CI rankings.

**Figure 4:**
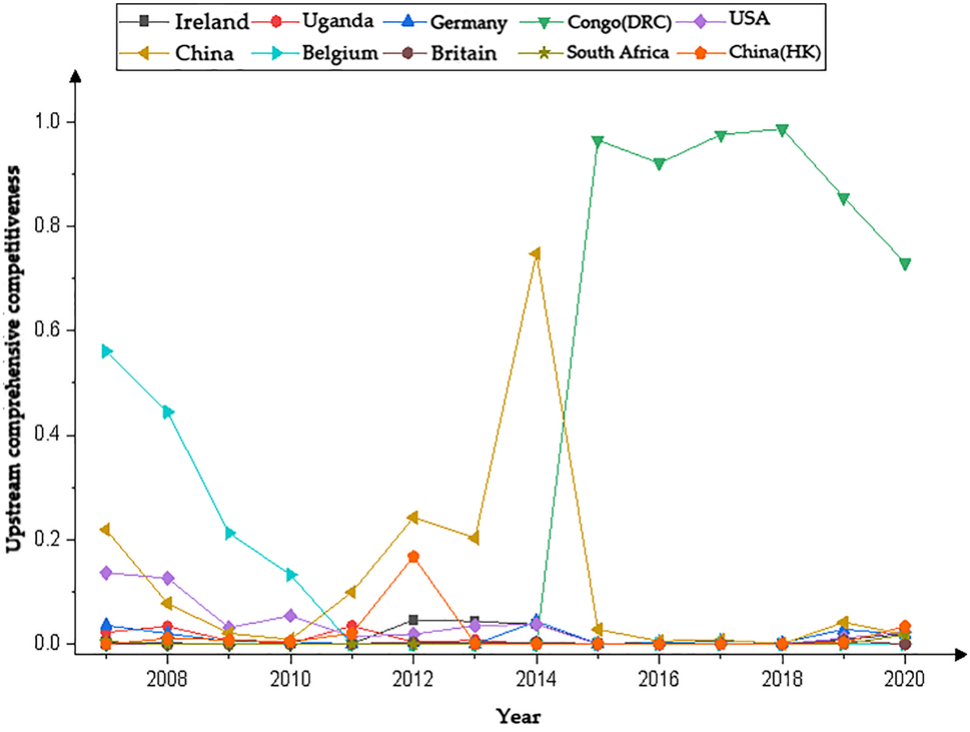
Changes in the upstream CI value.

**Figure 5:**
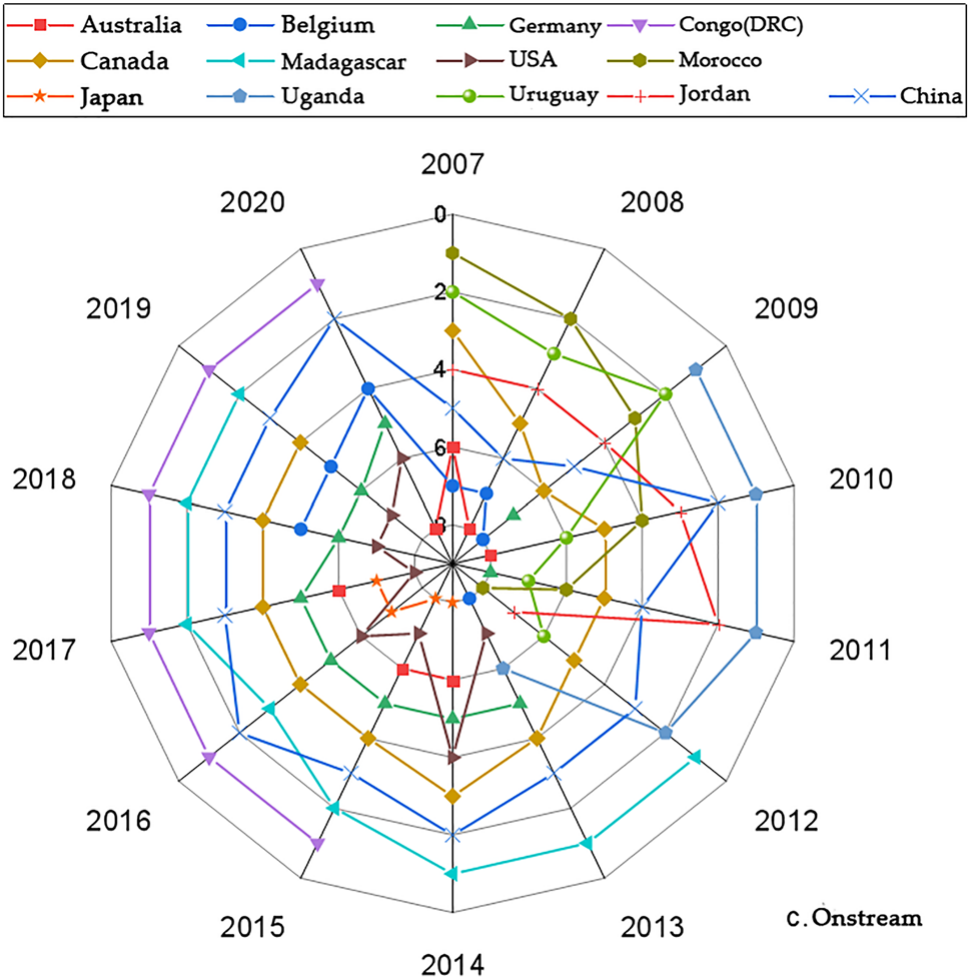
Changes in the midstream CI rankings.

**Figure 6:**
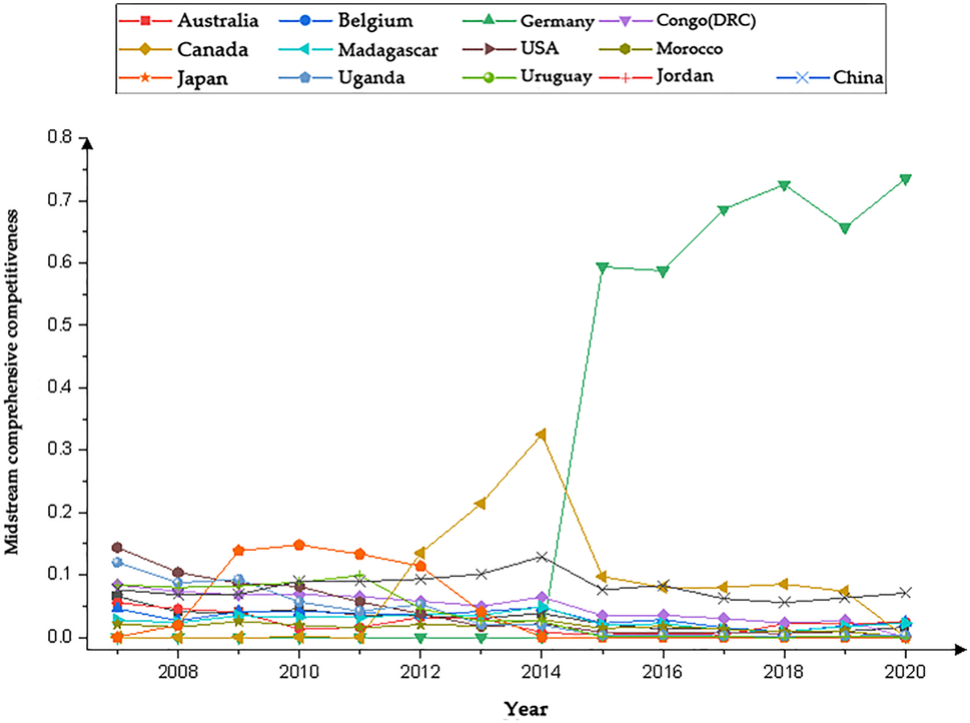
Changes in the midstream CI value.

**Figure 7:**
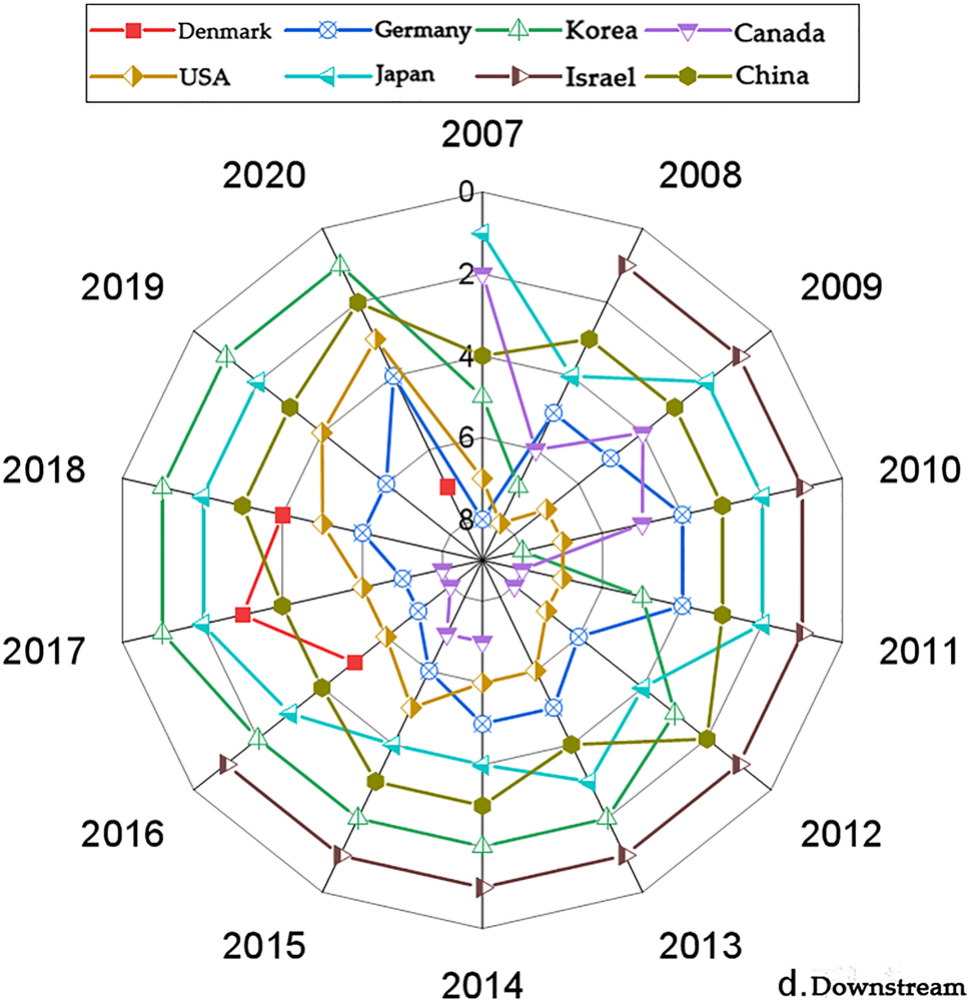
Changes in the downstream CI rankings.

**Figure 8:**
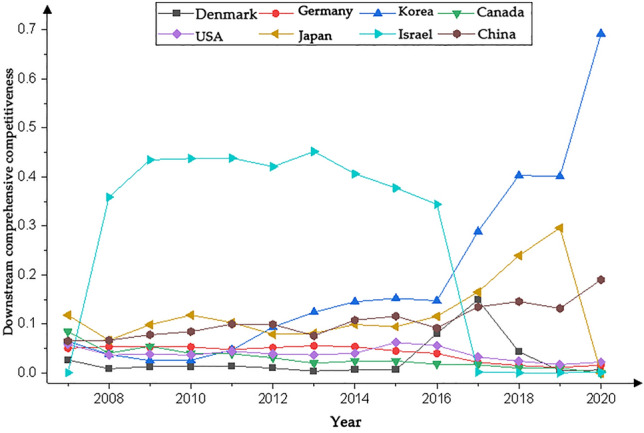
Changes in the downstream CI value.

We present the country with significantly higher rankings based on the networks generated by the Gephi software. Figures 1, 3, 5 and 7 represents the CI Index rankings for different countries. The year is displayed on the outside of the graphic, and the closer a country is to the center, the higher its ranking. Figure 2, 4, 6 and 8 shows the CI values.

#### Overall composite index analysis of the cobalt industry chain

The figures presented in Figs. 1 and 2 illustrate the rankings and fluctuations in the overall international competitiveness index (CI) of the global cobalt industry chain across different countries.

Figure 1 illustrates the CI index rankings of 12 countries, including the Democratic Republic of Congo (DRC), South Korea, and China, during the period of 2007–2020. The data indicate that these countries have consistently maintained high and stable rankings. Since 2015, the CI Index ranking of the Democratic Republic of the Congo (DRC) has consistently been the highest. The CI indices of South Korea, Japan, China, the U.S., and Germany have shown an overall fluctuating upward trend. Conversely, the rankings of Canada, Uruguay, Uganda, Israel, and Zambia exhibited an overall fluctuating downward trend. Rankings for Madagascar showed an initial upward trend and then a subsequent downward trend.

The CI index of the cobalt industry chain exhibited varying degrees of change in each country during the period of 2007–2020, as illustrated in Fig. 2. The CI Index of the Democratic Republic of the Congo (DRC) exhibited remarkable growth in 2015, surpassing that of other nations. Canada, Uruguay, Uganda, and Israel experienced a gradual decline in their CI scores. Conversely, South Korea, Japan, and China demonstrated an increasing trend, with South Korea's trend being particularly noteworthy. Following an initial upward trajectory, Germany and the United States experienced a decline before reaching their peak in 2014. Germany's CI exhibited more fluctuations than did that of the United States. Zambia and Madagascar experienced significant fluctuations, with Zambia displaying a rapid increase followed by a decrease and Madagascar showing a gradual upward and downward trend.

#### Analysis of the comprehensive index of the upstream cobalt industrial chain

The figures display the rankings and fluctuations in the comprehensive international competitiveness index (CI) of the upstream cobalt industry chain across various countries worldwide. Figures 3 and 4 correspond to the first and second positions, respectively. During the period from 2007 to 2010, there was a higher frequency of trade in upstream cobalt ores, and countries competed more intensely for this trade. However, from 2011 to 2020, the intensity of the competition gradually declined.

Figure 3 illustrates that ten countries (regions), such as the Congo (DRC), South Africa, and Germany, maintained a consistently high CI index ranking from 2007 to 2020. To enhance the analysis, China was included in the comparison. Since 2015, the CI has consistently ranked the Democratic Republic of the Congo (DRC) in the top position. Ireland and the United Kingdom have shown a fluctuating upward trend in their CI index rankings. The United States and Germany experienced a downward trend followed by an upward trend in their CI index rankings.

The CI of the upstream region of the cobalt industrial chain in each country (region) exhibited varying degrees of change during the period from 2007 to 2020, as illustrated in Fig. 4. Among these countries, the CI indices of the United States, Uganda, and Belgium exhibited a gradual decline. The CI index of Germany, the United Kingdom, Hong Kong, and China showed a pattern of initial decline followed by an increase. South Africa demonstrated a fluctuating trend of decline, followed by an increase and a subsequent decline. Ireland and the Democratic Republic of the Congo (DRC) displayed an initial increasing trend followed by a decreasing trend. The CI of the Democratic Republic of the Congo (DRC) exhibited remarkable growth in 2015, surpassing that of other countries by a significant margin. In contrast, the CI index of the U.S. displayed a fluctuating downward trajectory. Uganda's CI was notably higher during the 2007–2010 period but experienced a rapid and substantial decline, reaching a low level in 2011. Similarly, South Africa's CI index demonstrated a fluctuating pattern of decline, followed by an increase and subsequent decrease, reaching its peak in 2014. The CI index of China also exhibited a fluctuating trend, reaching its peak in 2014 while remaining consistently low.

#### Cobalt industry chain midstream composite index analysis

The figures depicting the rankings and fluctuations in the competitive index (CI) values of the midstream cobalt industry chain for various countries worldwide are presented in Figs. 5 and 6, respectively. Between 2007 and 2014, competition in the midstream cobalt ore trade intensified due to influences from the upstream cobalt ore trade. However, from 2015 onwards, competitive intensity decreased, and the rankings of countries in terms of competitiveness became more centralized and stabilized.

Figure 5 illustrates that thirteen countries, such as the DRC, Madagascar, and China, maintained a consistently high Corruption Perception Index (CI) ranking from 2007 to 2020. Since 2015, the Corruption Perceptions Index (CI) of the Democratic Republic of the Congo (DRC) has consistently held the top rank. The CI indices of Germany, Canada, the U.S., and China have exhibited fluctuating upward trends. Conversely, the rankings of Morocco and Uruguay have been declining. Additionally, the rankings of Madagascar, Japan, Uganda, and Jordan showed an initial upward trend followed by a subsequent decline, while the rankings of Australia and Belgium displayed a downward trend followed by an upward trend.

The CI of the midstream region of the cobalt industrial chain in each country (region) exhibited varying degrees of change during the period of 2007–2020, as illustrated in Fig. 6. The CI of the Democratic Republic of the Congo (DRC) exhibited significant growth in 2015, surpassing that of other countries. Madagascar, Uganda, and China demonstrated a pattern of initial increases followed by declines in their CI indices. Additionally, Madagascar and Uganda reached slightly higher CI index than did the other countries at their respective peaks, while China's CI index fell within the middle range, surpassing that of most listed countries, such as Australia, Belgium, and Germany. The majority of the remaining countries exhibit a similar pattern of change in their CI indices, characterized by an overall fluctuating downward trend and relatively comparable CI indices.

#### Comprehensive downstream index analysis of the cobalt industrial chain

The rankings and value changes of the competitive index (CI) for the downstream cobalt industry chain in various global countries are depicted in Figs. 7 and 8, respectively. During the period from 2007 to 2020, the competitive landscape of downstream cobalt mines became increasingly intense. Similarly, the competitiveness ranking of countries in the downstream sector appears to have gradually centralized and stabilized.

As can be seen in Fig. 7, during the period of 2007–2020, the CI rankings of eight countries, including South Korea, Japan, and China, are relatively high and stable. Among them, the CI index rankings of South Korea, the United States, Japan, and China show a fluctuating upward trend, with a larger increase in South Korea and the United States. The CI index rankings of Canada and Israel show a decreasing trend. Germany's CI index rankings show a rising and then decreasing trend, while Australia and Belgium show a decreasing and then increasing trend. Israel's CI index rankings were ranked first in the period of 2008–2016, but in 2017, the ranking began to plummet, reaching a low level. Denmark's CI index ranking was low in the previous period but has shown improvement in recent years.

As depicted in Fig. 8, the fluctuations in the CI index downstream of the cobalt industry chain differed from country to country between 2007 and 2020. Among them, Israel's CI index showed a trend of rising and then falling. The country's CI index level was the highest in 2008–2016, much higher than that of the other listed countries, and plummeted to a lower level after 2017. The CI indexes of South Korea, Japan, and China showed a fluctuating upward trend, with South Korea showing the highest level of CI indexes after 2017, and Japan and China also at a higher level. Germany, Canada, and the United States show a fluctuating downward trend in the CI Index. Denmark shows a downward, then upward, then downward trend.

### Benchmark model regression analysis

#### Stationarity test of variables

The stationarity test of variables is to avoid spurious regression problems in panel data.

We perform a unit root test on the data before estimating the model. This test helps ascertain the stationarity of the data. The results indicate that the initial sequence of all variables disproves the null hypothesis of a unit root at a significance level of 1%, allowing for the execution of panel regression.

Multicollinearity test of the independent variables. The Multicollinearity test of the independent variables is to avoid the issue of regression result inaccuracy stemming from multicollinearity among the independent variables.

We performed a variance-inflated factor analysis of the regression equation. The results indicate that the variance expansion factor of each variable is less than 10, suggesting the absence of multicollinearity among the variables.

Benchmark regression. The benchmark regression was conducted to test the impact of cobalt resources trade network characteristics on the international competitiveness of industries.

Before the model was implemented, the Hausman test was conducted, indicating that the fixed effect model (FE) should be selected and the year utility adjusted. Table 3 provides a thorough analysis of the impact of the unique characteristics of the cobalt resource trade network on the global competitiveness of the industry. The examination of models (1) through (8) indicates that the characteristics of a country's cobalt resource trade network have a significant impact on the sector's global competitiveness. The significance of network centrality and network heterogeneity in bolstering industrial international competitiveness is underscored, confirming hypotheses 1 and 3. Conversely, the strength of network connections exerts a notable inhibitory effect on the enhancement of industrial international competitiveness, thus confirming hypothesis 2. After controlling for other variables, a 1 percentage point increase in a country's weighted eigenvector centrality results in a 0.000137 percentage point increase in the international competitiveness of its cobalt resource industry. Likewise, a 1% increase in the strength of a country's cobalt resource trade network leads to a 0.000108% decrease in the international competitiveness of its cobalt resource industry. Moreover, a 1% increase in the weighted effective scale of a country's cobalt resource trade network results in a 0.000267% increase in the international competitiveness of its cobalt resource industry.

**Table 3:** Regression results of the benchmark model.

explanatory variables	(1)	(2)	(3)	(4)	(5)	(6)	(7)	(8)
$InWeigcentrality$	0.000295***			0.000147***	0.000146***	0.000157***	0.000152***	0.000137***
	(0)			(0.000171)	(0.000158)	(7.07e−05)	(0.000106)	(4.84e−05)
$InWoutdegree$		− 4.20e−05**		− 0.000122***	− 0.000122***	− 0.000121***	− 0.000116***	− 0.000108***
		(0.0465)		(3.15e−06)	(3.98e−06)	(5.26e−06)	(8.47e−06)	(8.52e−07)
$Ineffecsize$			0.000785***	0.000642***	0.000639***	0.000655***	0.000677***	0.000267***
			(0)	(3.81e−10)	(1.01e−09)	(2.37e−10)	(9.04e−11)	(0.00595)
$Infintinvetment$					− 2.39e−05	− 1.78e−05	− 5.06e−05	− 0.000193***
					(0.517)	(0.629)	(0.164)	(1.80e−06)
$Indomprisector$						− 0.000102*	− 8.12e−05	− 7.61e−05
						(0.0951)	(0.192)	(0.143)
$InGDPgrow$							0.000271***	0.000226***
							(0.000873)	(0.00620)
$InExdependency$								0.000364***
								(0)
constant term	1.001***	1.000***	1.000***	1.000***	1.000***	1.000***	1.000***	1.002***
	(0)	(0)	(0)	(0)	(0)	(0)	(0)	(0)
time effect	1	1	1	1	1	1	1	1
sample	871	871	871	871	871	871	871	871
model	FE	FE	FE	FE	FE	FE	FE	FE
Adjusted R^2^	0.190	0.025	0.182	0.248	0.249	0.252	0.266	0.384

The values in parentheses represent standard errors. * * * , * * and * indicate that the coefficients passed the significance test at 1%, 5% and 10%, respectively. The same applies below.

The weighted eigenvector centrality within the cobalt resources trade network can serve as an indirect indicator of a country's influence, as it reflects the quantity and quality of its cooperative relationships with other countries. The level of international competitiveness within a nation's cobalt resources industry is directly correlated with the weighted eigenvector centrality, which is, in turn, impacted by the caliber of countries collaborating with the nation. This occurs due to collaboration with developed nations, which enables the acquisition of technological knowledge and skilled personnel through the imitation effect and the learning-by-doing effect. It also facilitates the efficient utilization of the nation's cobalt resources in the global division of labor, thereby bolstering the industry's international competitiveness. The out-strength metric indicates the degree to which a nation's cobalt resources are being exported. Given that cobalt resources are nonrenewable, a higher out-strength suggests a reduced level of international competitiveness in the country's cobalt resources industry. As the weighted effective scale degree increases, the nonredundancy level of the node also increases, resulting in greater network heterogeneity. As a result, the global competitiveness of the industry has increased.

Furthermore, the control variables have negative coefficients for FDI and positive coefficients for GDP growth and trade openness, all of which are statistically significant at the 1% level. This aligns with the anticipated outcomes. The excessive influx of foreign individuals may result in their dominance of the domestic consumer market, potentially impeding trade relations between the host country and other nations. Hence, foreign direct investment has a notable inhibitory impact on the international competitiveness of a nation's cobalt resource industry in the present phase. The influence of financial development on the international competitiveness of the cobalt industry appears to be limited, likely due to the predominance of state capital in this industry. The ratio of private capital's credit scale to the overall credit scale in the country is inaccurate for accurately assessing the industry's impact based on the level of financial development. The impact of GDP growth on the international competitiveness of the industry is significant. GDP growth can facilitate trade expansion, thereby enhancing the international competitiveness of the key cobalt resources industry for a country. A higher level of trade openness in a country is associated with reduced trade barriers and transaction costs, thereby enhancing the international competitiveness of its industries.

### Endogeneity test

The endogeneity test is designed to address the problem of two-way mutual causation, between different variables.

Enhancements in the global competitiveness of a nation's cobalt resource sector could lead to an expansion in cobalt trade with other nations, and this increased trade, in turn, contributes to the enhancement of the sector's global competitiveness. Therefore, there may be a bidirectional causality between them, in other words, endogeneity.

This paper utilizes the two-step system generalized method of moments (GMM) estimation to addressing endogeneity. This method is less affected by heteroskedasticity compared to one-step estimation, leading to consistent and effective GMM estimates. Consequently, it can enhances the accuracy and efficiency of the estimation results^51,52^.

Firstly, the lagged term of the network centrality indicator is chosen as an instrumental variable. Secondly, we utilize SPSS software to conduct a two-step GMM estimation to re-test the panel data. Table 4 displays the results of the two-step GMM estimation approach. Finally, the results of the Adjusted R^2^ test and Hansen J test *p*-value are both greater than 0.1, indicating that the initial hypothesis cannot be refuted. This confirms the validity of the instrumental variables and supports the original hypothesis that there is no autocorrelation in the error terms. Consequently, the validity of the two-step GMM estimation method is confirmed, and the estimation results are consistent with the benchmark model regression results above without significant endogeneity.

**Table 4:** Two-step GMM estimation results.

variables	(9)	(10)
$InWeigcentral$	0.000173***	0.000166***
	(0.000241)	(3.77e−05)
$InWoutdegree$	− 0.000154***	− 0.000131***
	(6.10e−05)	(6.75e−05)
$Ineffecsize$	0.000618***	0.000268**
	(1.02e−06)	(0.0237)
$Infintinvetment$		− 0.000185***
		(1.29e−05)
$Indomprisector$		− 2.41e−05
		(0.649)
$InGDPgrow$		0.000204**
		(0.0183)
$InExdependency$		0.000309***
		(0)
Constant term	1.000***	1.002***
	(0)	(0)
Hansen J test	0.5721	0.7508
Sample	737	737
Adjusted R^2^	0.271	0.379

The values in parentheses represent standard errors. * * * , * * and * indicate that the coefficients passed the significance test at 1%, 5% and 10%, respectively.

### Robustness test

The robustness tests are conducted to verify the non-randomness and reliability of the study results.

To assess the reliability of the regression findings in this study, a robustness analysis was performed. This analysis involves the utilization of both changed explanatory variables and panel quantiles. The first step requires replacing explanatory factors to assess the influence of a country's cobalt resources trade network features on the global competitiveness of industries. The competitive advantage index (CA index) refers to the difference between the relative proportion of a country's and the global exports of a certain product and the relative proportion of their imports of that product. In contrast to the RCA and TC indices, the CA index considers both the import trade situation and the economic size status of a country. However, this approach lacks the ability to differentiate between various regions and market shares. Therefore, the CA index can be employed in lieu of the composite index (CI) for conducting a robustness test. The regression findings for the specific variables can be found in models (11) and (12) in Table 5. The findings indicate that the robustness test results obtained by substituting the explanatory variables align with the benchmark regression results. Moreover, it is observed that both network centrality and network heterogeneity significantly contribute to enhancing industrial international competitiveness. Conversely, network connectivity has a notable inhibitory effect on the improvement of industrial international competitiveness when other variables are controlled for. In general, the results of this study demonstrate a certain degree of robustness.

**Table 5:** Robustness test results.

Variable	(11)	(12)	(13)	(14)	(15)	(16)	(17)
	InCA	InCA	10%	20%	50%	70%	85%
$InWeigcentral$	0.00409***	0.00416***	1.72e−05***	1.92e−05***	3.84e−05***	4.88e−05	9.11e−05*
	(0)	(0)	(6.83e−08)	(3.26e−05)	(0.00548)	(0.113)	(0.0925)
$InWoutdegree$	− 0.00371***	− 0.00352***	− 5.91e−06***	− 5.19e−06**	− 3.28e−05***	− 0.000101***	− 0.000206***
	(1.04e−10)	(0)	(0.000194)	(0.0106)	(0.000141)	(0.000120)	(2.57e−05)
$Ineffecsize$	0.0126***	0.00616***	3.81e−05***	4.68e−05***	0.000116***	0.000256***	0.000508***
	(1.00e−10)	(1.26e−05)	(9.34e−09)	(1.54e−10)	(0.000460)	(0.00136)	(0.00860)
$Infintinvetment$		− 0.000586**	− 8.16e−07	− 4.78e−06	− 3.83e−05**	− 0.000113**	− 0.000275***
		(0.0391)	(0.825)	(0.270)	(0.0272)	(0.0228)	(0.00839)
$Indomprisector$		− 0.00165*	− 6.58e−07	− 4.34e−06	− 1.91e−05	− 3.66e−05	− 0.000130
		(0.0627)	(0.880)	(0.510)	(0.275)	(0.451)	(0.188)
$InGDPgrow$		0.00256***	− 8.69e−06	− 1.22e−06	2.57e−05	6.12e−05	0.000255
		(0.00494)	(0.110)	(0.821)	(0.207)	(0.338)	(0.157)
$InExdependency$		0.00650***	4.98e−05***	5.83e−05***	9.95e−05***	0.000181***	0.000387***
		(0)	(0)	(0)	(1.92e−10)	(3.58e−05)	(1.49e−10)
Constant term	1.971***	2.011***	1.000***	1.000***	1.001***	1.001***	1.002***
	(0)	(0)	(0)	(0)	(0)	(0)	(0)
Sample	871	871	871	871	871	871	871

The values in parentheses represent standard errors. * * * , * * and * indicate that the coefficients passed the significance test at 1%, 5% and 10%, respectively.

The explanatory variables can be used to assess the reliability of the benchmark regression, which measures the influence of a country's cobalt resource trade network characteristics on the improvement of industries' international competitiveness. However, this approach fails to reveal the relationship between changes in trade network characteristics and the international competitiveness of industries. To address this, the paper employs panel quantile regression to further confirm the reliability of the findings. The specific outcomes are presented in Table 5. The regression findings of models (13)–(17) demonstrate that the panel quantile regression method is used to analyze the factors that influence the international competitiveness of sectors with varying conditional distributions. As a country's cobalt resources industry transitions from the lower to the upper echelons of the conditional distribution in terms of international competitiveness, the influence of the country's trade network characteristics on the industry's global competitiveness undergoes distinct changes. Specifically, the positive impact of network centrality and network heterogeneity on bolstering the industry's international competitiveness increases, while the negative effect of the strength of network linkages on enhancing the industry's global competitiveness gradually diminishes.

## Conclusions and discussion

### Conclusion

This paper develops a comprehensive evaluation index system for the international competitiveness of the cobalt resources industry based on theories of comparative advantage and competitive advantage. The comprehensive index is measured using global cobalt trade data from 2007 to 2020, and the evolution of the international competitiveness of the entire cobalt industry chain, including its upstream, midstream, and downstream sectors, is empirically analyzed. Additionally, a panel regression model is constructed to explore the relationship between the characteristics of the cobalt resource trade network and industry competitiveness. The study finds the following:

1. The demand for different products of the cobalt industrial chain in countries worldwide has changed: From the point of view of the degree of trade competition at each stage of the cobalt industrial chain, with the development of the cobalt resource trade, in recent years, the focus of trade competition for cobalt resources in various countries has changed from upstream to midstream to the middle to lower reaches of trade, and the trade competition for cobalt resources in the lower reaches of trade has become increasingly intense, and the main body of the competition is the Republic of Korea, Japan, China, and other regions.
2. Competition in the cobalt industry chain has gradually turned into national centralization and stabilization from early multicountry competition: From the perspective of the international competitiveness level of cobalt resources in global countries, and the international competitiveness of each country at different stages of the cobalt industry chain has varied. As far as China is concerned, affected by upstream cobalt resource reserves, China's international competitiveness level upstream of the cobalt industry chain is relatively low, but the international competitiveness level embodied in the middle and lower reaches of the industry chain is relatively high; thus, the international competitiveness level of the industry chain as a whole is also relatively high.
3. Network centrality and network heterogeneity significantly promote industrial international competitiveness: As can be seen from the regression analysis, , and this effect strengthens as industries move from low to high international competitiveness. Moreover, strong network ties significantly inhibit industrial international competitiveness, with this effect weakening as international competitiveness changes. The control variable, foreign direct investment, inhibits the international competitiveness of industries, while the coefficients of GDP growth and trade openness promote international competitiveness.

## Discussion

The above findings indicate that countries worldwide are actively participating in the trade network of essential nonferrous metals. However, there is a disparity in the international competitiveness of the nonferrous metals industry across different countries. Hence, in the context of globalization, if China aims to leverage its trade advantages to enhance the international competitiveness of the key nonferrous metal industry and strengthen control over the industry chain, it must investigate the trade cooperation opportunities and potential of key nonferrous metals at all stages of the industry chain within the current network trade pattern. Consequently, for China the following suggestions are proposed:

First, the "breadth" and "depth" of trade should be enhanced. China ought to enhance its trade in cobalt resources, with countries exhibiting a higher weighted degree of centrality. This involves reinforcing downstream cobalt resource trade with Canada and upstream cobalt resource trade with South Africa. Additionally, it is imperative for us to assume a significant role as a transit country for trade with nations possessing more influential intermediary centers, thereby enhancing China's capacity to regulate resources and information intermediaries within the global trade network. Moreover, it is imperative to engage in trade with other nations or regions that possess high heterogeneity. This involves bolstering the trade of downstream cobalt resources with South Korea and Japan on the current foundation, thereby indirectly amplifying China's cobalt resources industry influence through the technological advantages and influence of these countries.

Second, the industrial chain should be extended while emphasizing diversification on the supply side to ensure supply security. Upstream of the industrial chain, China needs to maintain its cooperation with the Congo (DRC) and, at the same time, increase the trade of upstream cobalt raw materials with Belgium, Zambia, South Africa, and Ireland to diversify the import of cobalt resources to ensure the security of the supply at the upstream end. In the middle of the industrial chain, we should emphasize cobalt resource trade with Britain and Japan, maintain trade with Tanzania, New Caledonia, and Russia, and limit export trade with Australia and Canada to guarantee adequate supply in the middle. Downstream of the industrial chain, it is still necessary to pay attention to trade with Korea and Japan while strengthening the domestic production of lithium cobaltate; increasing the export trade of downstream cobalt resources with Germany, the United Kingdom, and Canada; and increasing import trade with Poland, Canada, Vietnam, Austria, and other countries.

The above policy recommendations are clearly based on a more inclusive and open trade environment. Cobalt, being a strategically important metal, may face obstacles in future raw material exports, particularly from Africa, due to ongoing global conflicts. As a result, China may further expand its influence in the African region, thereby securing its supply of upstream cobalt raw materials.

## Data Availability

The data that support the findings of this study are available from UN Comtrade database and World Bank (WDI) database but restrictions apply to the availability of these data, which were used under license for the current study, and so are not publicly available. Data are however available from the corresponding author upon reasonable request and with permission of UN Comtrade and World Bank (WDI).
